# Unexpected death after headache due to a colloid cyst of the third ventricle

**DOI:** 10.1186/1477-7819-4-47

**Published:** 2006-07-25

**Authors:** Sameer S Shaktawat, Walid D Salman, Zuhair Twaij, Abdul Al-Dawoud

**Affiliations:** 1 Department of Cellular Pathology, Blackpool Victoria Hospital, Blackpool, Lancashire, UK; 2 Department of Histopathology, Burnley General Hospital, Burnley, Lancashire, UK

## Abstract

**Background:**

Colloid cysts of the third ventricle are rare benign intracranial non-neoplastic cysts. Headache is the most common symptom. We present a case of sudden death due to colloid cyst in a 17 year old female who had symptoms of intermittent headaches.

**Case presentation:**

A 17 year old female presented with intermittent mild headaches for a period of two years. She complained of severe headache in the night and was found unresponsive the next morning. Resuscitation team was called on site but the patient was already dead. At post mortem examination a dilated ventricular system was found with a colloid cyst of the third ventricle.

**Conclusion:**

This report highlights the difficulty in the diagnosis and importance of recognizing colloid cyst of the third ventricle which should be in the differential diagnosis of headaches in children and young adults and of hydrocephalus at autopsy

## Background

Colloid cysts of the third ventricle are a rare cause of headache and sudden death. Approximately three persons per million per year are affected by this entity [1]. We present a case that was only discovered at autopsy, but that on reflection displayed classical clinical features. Colloid cysts may present in various clinical settings ranging from intermittent headaches to non-specific symptoms and uncommonly as sudden deaths. Cases of sudden death due to colloid cysts could be explained by the non-recognition of the symptoms especially if they are mild and non specific. Although classically presenting during the 3^rd ^to 5^th ^decades, rare cases of colloid cysts in children and young individuals have been reported. Magnetic resonance imaging (MRI) and computed tomography (CT) may be used for a preoperative diagnosis. Although the colloid cyst can show variable signal characteristics, the clinical features with their classical location can be helpful in the early and correct diagnosis of this benign but potentially fatal entity.

## Case presentation

**Figure 1:**
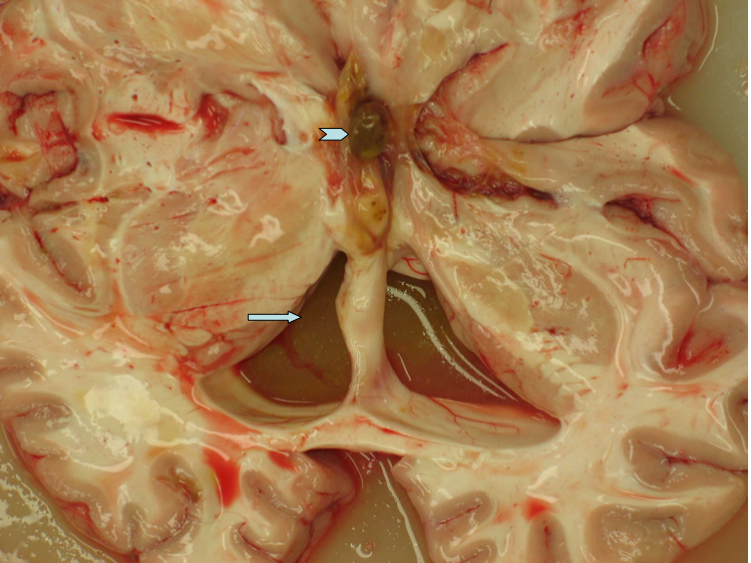
Gross picture of the brain, arrow shows dilated ventricle due to blockage of the foramen of Monro by a colloid cyst (chevron).

A 17 year old female presented with intermittent headaches for a period of about two years. The symptoms were considered too mild and no medical opinion was taken. Over the counter medicines were used during this period for the headaches which came back at regular intervals. She complained of mild headache in the night and was found in her bed next morning, unresponsive, not breathing and without a pulse. The ambulance and resuscitation team reached in ten minutes but could not revive the patient. Post mortem examination was conducted. External examination was unremarkable and there was no evidence of any injury or trauma. The main findings were in the central nervous system which showed a dilated ventricular system. This appeared secondary to blockage of the third ventricle by a brownish yellow colloid cyst measuring 1 cm. in maximum dimension (Figure 1). The area surrounding the cyst showed greenish-brownish fluid. The cerebral cortex appeared generally edematous. Both lungs showed intrapulmonary hemorrhage associated with congestion and moderate edema. All the other organ systems were normal and showed no abnormalities.

## Discussion

Colloid cysts qualify as non-neoplastic true epithelium lined cysts of the central neuraxis. These are smooth, round lesions of endodermal origin and are located at the antero-superior aspect of the third ventricle. They generally present in the 3^rd^–5^th ^decade but they can present at both extremes of ages. They usually arise from the inferior aspect of septum pellucidum and protrude into the anterior portion of the third ventricle between columns of fornix. Cyst size can range from 3–40 mm but size may not be a reliable predictor of outcome as even small cysts may cause sudden death [2]. There are reports of colloid cysts at unusual locations such as lateral ventricle and posterior fossa. They generally present with manifestations of ventricular outflow obstruction, a consequence of their location at the foramen of Monro in the anterior aspect of the third ventricle. The primary presenting complaint is headache. The headaches are classically intermittent, episodic, sometimes intense and severe. This headache is decreased on lying down which is unusual for a headache due to an intracranial space occupying lesion. Associated symptoms include vertigo, memory deficit, diplopia and behavioral disturbances. The clinical finding of headaches is due to transient obstruction secondary to ball valve mechanism at the foramen of Monro. The mechanism(s) of death in colloid cyst is a controversial subject but some authors have tried to elucidate this mechanism, indicating it to be a multifactorial and dynamic process. The acute deterioration is possibly initiated by increase in sagittal sinus pressure, which provokes acute brain swelling and ultimately a series of events leading to death [3]. The symptoms can be so subtle as to escape the diagnosis and possible therapeutic intervention. There are instances when the patient remains asymptomatic and sudden death in such patients has been reported [4]. Some authors report that there is a likelihood of autosomal dominant inheritance though the genetic aspect has not been clearly studied, but still the plea is made for a detailed family history of relatives in such cases [5].

Histologically colloid cysts are lined by cuboidal, pseudostratified or columnar and mucus-secreting epithelial cells. The cyst contains mucoid and gelatinous material which is Periodic acid Schiff (PAS) positive. Occasionally squamous metaplasia, hemosiderin pigment and xanthogranulomas are seen in the cyst wall [6]. Colloid cysts can be diagnosed on computed tomography (CT) or MRI. On nonenhanced CT, the majority of the cysts are hyperdense. On MRI the cysts are hyprintense on both T1 (longitudinal relaxation time) and T2 (transverse relaxation time) weighted sequences in more than half of the patients [7]. Stereotactic brain fine needle aspiration (FNA) is a useful diagnostic tool for evaluating space occupying lesions especially colloid cysts. The FNA shows radiate hyphae-like structures which are often admixed with liquid contents of the cyst, for practical purposes is diagnostic of colloid cysts [8]. Cytomorphology with radiologic features and clinical correlation is sufficient for the diagnosis of this rare pathologic entity. Therapeutic options include stereotactic cyst aspiration, ventriculoperitoneal shunts, transcortical, transcallosal resection and endoscopic removal and marsupialization. Radical removal by open or stereotaxically guided microsurgery is the method of choice in some cases with stereotaxic microsurgical laser craniotomy with or without biventricular shunting also been described [1,8]. Differential diagnoses from the same site located masses include meningiomas and ependymomas. The pulmonary oedema in our case can be explained on the basis of a study by Smith and Matthay which described neurogenic pulmonary oedema, characterised by an "increase in extravascular lung water in patients who have sustained a change in neurological condition" [9]. Both high-pressure and increased-permeability abnormalities may be involved in the pathogenesis of pulmonary edema in such conditions [10].

## Conclusion

To summarize, this case report is an attempt to remind clinicians that colloid cysts although rare should remain in the differential diagnosis of headache in children and healthy young adults. It should also remind pathologists that colloid cysts are a rare cause of hydrocephalus present at autopsy. Recognition of the clinical findings in colloid cyst and subsequent CT or MRI may result in early diagnosis and decreased mortality.

## Competing interests

The author(s) declare that they have no competing interests.

## Authors' contributions

**SSS**: Drafting of the manuscript and did a thorough research of the medical literature on the subject, **WDS**: Contributions to conception and design, **ZT**: Intellectual contributions and provided with scientific input, **AAD**: Final approval to the manuscript. All the authors read and approved the final manuscript.
